# A two-step structural equation modeling and explainable machine learning framework for understanding university students’ adoption of generative AI: balancing intrinsic motivations and perceived risks

**DOI:** 10.3389/fpsyg.2026.1743722

**Published:** 2026-01-29

**Authors:** Daixing Zeng, Xiaoqin Xu, Tianxing Zhu, Yong Li, Qiumin Li

**Affiliations:** 1 Department of Statistics, Chengdu University of Information Technology, Chengdu, China; 2 Jinan University-University of Birmingham Joint Institute, Jinan University, Guangzhou, China; 3 School of Mathematics, Southwest Jiaotong University, Chengdu, China; 4 Key Open Laboratory of Statistical Information Technology and Data Mining, National Bureau of Statistics of China, Chengdu, China

**Keywords:** 2SSX research framework, benefit–risk duality, explainable machine learning, GenAI adoption, technology acceptance model

## Abstract

The adoption of generative AI tools by university students has surged, embodying a mix of promising benefits and serious concerns. Understanding the factors that drive or hinder students’ adoption of GenAI is essential for responsible integration of AI technologies in higher education. This study introduces a novel two step SEM–XML framework that couples structural equation modeling (SEM) with an explainable machine learning (XML) component, overcoming limitations of traditional SEM and enabling both hypothesis-driven path analysis and data-driven factor identification. Grounded in an integrated benefit–risk perspective, this framework blends constructs from the Technology Acceptance Model, Theory of Planned Behavior, Perceived Risk, and Knowledge Attitude Practice models, emphasizing students’ intrinsic motivations. The study is designed as a cross-sectional survey, with an effective sample size of 880 respondents from southwestern China, including undergraduate, master’s, and doctoral students. The average age of participants is 20.8 years, with a gender distribution of 48.52% male and a diverse academic background, encompassing fields such as Engineering, Economics, Science, and Management. We test this framework using a survey of university students’ GenAI usage. Results show that positive perceptions such as perceived usefulness and personal interest strongly encourage GenAI use. In contrast, perceived risks related to ethics, accuracy, and academic integrity significantly inhibit it. This pattern is partially consistent with previous findings on ChatGPT adoption. These findings highlight how internal attitudes and external pressures interact to shape GenAI uptake. This study emphasizes the substantial impact of both internal and external factors on students’ acceptance of GenAI tools, providing valuable insights for educational institutions, policymakers, and tool developers.

## 1 Introduction

The adoption of Generative Artificial Intelligence (GenAI) in higher education is transforming learning and teaching processes. Li et al. (2019) highlighted how emerging technologies, such as AI and blockchain, enhance student engagement and outcomes. Tools like ChatGPT, for instance, support personalized learning by generating content and assisting with problem-solving (García-Alonso et al., 2024). Kiryakova and Angelova (2023) pointed out that GenAI has the potential to revolutionize education, particularly when educators utilize it to enhance instructional efficiency. However, Hasanein and Sobaih (2023) warned that educators must strike a balance between the benefits of GenAI and concerns about academic integrity and cognitive development. Stöhr et al. (2024) stressed that the adoption of GenAI varies across disciplines, suggesting that integration strategies should be tailored to each field. In a similar vein, Naidoo (2023) explored how the integration of AI with blockchain can enhance e-learning engagement and performance, emphasizing the role of perceived usefulness and ease of use in promoting adoption.

Recent research on GenAI adoption in education has focused on three primary themes: usage patterns, driving factors, and challenges related to academic integrity (Baek et al., 2024; Brown et al., 2020; Chan, 2023). First, usage patterns: Many studies have explored how frequently and in what contexts students use GenAI tools. Deng et al. (2024) found that GenAI is widely employed for tasks such as information retrieval, content generation, coding assistance, and language polishing. Stöhr et al. (2024) noted that usage patterns differ across disciplines—humanities students tend to use GenAI for literature reviews and writing improvement, while students in science and engineering fields often use it for programming and data simulation. Undergraduates increasingly rely on GenAI for thesis writing, while graduate students use it for drafting research papers. These patterns raise concerns about academic standards and the potential for over-dependence on AI tools (Hasanein and Sobaih, 2023). Second, driving factors: Researchers have applied established technology acceptance frameworks, such as TAM and UTAUT, to explain GenAI adoption. Saif et al. (2024), Albayati (2024), and Rahul et al. (2023) identified core constructs like perceived usefulness, ease of use, self-efficacy, peer influence, and institutional support as key drivers of behavioral intention and actual usage. Zarifis and Efthymiou (2022) highlighted the importance of aligning AI adoption with business models in education, suggesting that institutions should adopt AI in ways that complement their specific needs and capabilities. However, Wang et al. (2024) found significant barriers, such as concerns about plagiarism and the difficulty of distinguishing AI- from human-generated content. These concerns were echoed by Shamsuddinova et al. (2024), who argued that over-reliance on GenAI could diminish students’ critical thinking abilities. To address these challenges, Rahul et al. (2023) stressed the need for clear institutional policies, while Alqahtani and Wafula (2025) emphasized the importance of training programs to ensure the ethical use of AI tools and prevent misuse. Third, academic integrity and assessment challenges: The rise of GenAI has raised questions about the fairness and effectiveness of student evaluation. Cotton et al. (2024) noted the difficulty of distinguishing AI-generated text, as sophisticated paraphrasing can evade traditional plagiarism detectors. The boundaries between legitimate use (e.g., drafting support) and academic dishonesty are increasingly blurred. Scholars suggest adapting teaching and evaluation methods, such as using open-ended questions, emphasizing the writing process, and incorporating oral assessments, to ensure GenAI is used as a complement to learning rather than a substitute (Stone, 2023; Karkoulian et al., 2024).

The perceived risks of GenAI adoption are central to its integration into education. Alqahtani and Wafula (2025) highlighted ethical risks and the potential for academic misconduct. Ravšelj et al. (2025) noted that while GenAI can enhance productivity, it may also lead to dependency, reducing student engagement. Saif et al. (2024) emphasized the challenge of balancing innovation with academic integrity, and Tao et al. (2019) and Jablonka et al. (2024) stressed the need to align AI use with institutional goals. Shankar et al. (2023) found some students hesitant to adopt GenAI due to concerns about losing critical thinking skills. Finally, Venkatesh et al. (2012) and Teo and Noyes (2011) extended the TAM framework, suggesting that GenAI tools must be both useful and engaging for long-term adoption. Rughiniș et al. (2025) also discussed the role of institutional policies in managing the adoption of AI technologies, emphasizing that universities must find ways to regulate AI use while maintaining academic values and integrity.

Research on institutional factors further underscores the role of external pressures on students’ decisions. Albayati (2024) pointed out that students’ attitudes toward AI tools are shaped by institutional guidelines and educational culture. Hamd et al. (2023) emphasized the need for institutional support to guide both students and faculty. Shamsuddinova et al. (2024) stressed that responsible adoption depends on the interaction between students’ intrinsic motivations and institutional regulations. Prentzas (2013) argued that GenAI tools should complement traditional educational goals, while Alqahtani and Wafula (2025) suggested fostering a culture of innovation with proper ethical training for students and faculty.

Another critical aspect of GenAI adoption is technological literacy. Tao et al. (2019) found that students’ understanding of GenAI tools, such as large language models, directly affects their engagement. Al-Azawei et al. (2017, 2019) emphasized that students with higher technological literacy are better able to evaluate the quality of AI-generated content. Morris (2023) stressed the importance of fostering digital literacy to address concerns about misinformation and bias in AI outputs. Scherer et al. (2019) noted that students with prior AI experience are more open to adopting new AI tools. Thus, improving digital and AI literacy is crucial for effective integration of GenAI in education (Milano et al., 2023; Mittal et al., 2024).

While these studies provide a solid foundation, there are still limitations. Few studies have integrated benefits, risks, and cognitive theories to examine the adoption of GenAI in higher education, and even fewer have concretized the influencing factors through post-path analysis. Many existing studies rely on the Technology Acceptance Model (TAM) and related frameworks, but they often overlook education-specific factors, potentially missing the subtle dynamics within learning contexts (Bouebdallah and Youssef, 2025). Given the innovative capabilities of generative AI, it is essential to consider both its perceived benefits and risks within educational settings. Moreover, SEM is typically used to analyze direct, indirect, and moderating relationships between latent variables, but it lacks a deeper examination of measured variables. To address these limitations, this study introduces the “2SSX” (two-step SEM–XML) research framework. In the first stage, we apply SEM to test the hypothesized relationships between students’ motivations, attitudes, and intentions. In the second stage, we use explainable machine learning techniques to identify key factors, shifting from subjective hypothesis testing and latent-variable path analysis to objective, measured variables insights.

The potential contributions of this study are threefold:

1. Surveying and understanding the overall adoption of GenAI by university students, providing valuable insights for related research.
2. Developing a dual-benefit–risk theoretical model of GenAI adoption based on a synthesis of existing studies. This model integrates key frameworks such as the Technology Acceptance Model, the Theory of Planned Behavior, Perceived Risk Theory, and the Knowledge-Attitude-Practice Model. Using this model, we empirically test the hypotheses via SEM.
3. Introducing the 2SSX framework, which combines SEM with explainable machine learning techniques. This dual-stage approach not only tests theoretical paths but also identifies key factors at a granular level. The 2SSX framework offers additional insights beyond SEM, helping visualize core influencing factors and providing both methodological reference and empirical evidence for future studies.

## 2 Theoretical framework and research hypotheses

### 2.1 Extended technology acceptance model

#### 2.1.1 Technology acceptance model (TAM) and theory of planned behavior (TPB)

The concept of technology acceptance originated with Fishbein and Ajzen’s Theory of Reasoned Action (TRA) in 1975, which defines it as an individual’s intention to adopt a certain technology to accomplish specific tasks. The fundamental assumption of the theory is that users’ beliefs influence their behavioral intentions, and these intentions, in turn, determine their actual behavior toward using a given technology (Taylor and Todd, 1995).

Building on TRA, Ajzen proposed the TPB, which posits that an individual’s behavioral intention is influenced by three key factors: attitude toward the behavior, subjective norm, and perceived behavioral control. The more positive one’s attitude, the stronger the perceived social norms and behavioral control, and the stronger the behavioral intention—ultimately increasing the likelihood of performing the behavior (Ajzen, 1991). TPB emphasizes that behavioral intentions are not formed arbitrarily but are shaped by personal attitudes, social expectations, and perceived control. Subjective norms play an essential role in decision-making, as individuals often experience social pressure when determining whether to engage in a particular behavior.

Davis (1989) later applied TRA and TPB to the field of information systems to explain users’ acceptance of information technologies, thus developing the TAM. TAM explains the factors influencing an individual’s adoption of a particular technology within an organizational or social context (Davis, 1989). It identifies two core determinants of technology adoption intention: perceived usefulness (PU) and perceived ease of use (PE) which jointly determine users’ behavioral intentions.

Perceived usefulness refers to a potential user’s subjective evaluation of the extent to which using a particular technology or product will enhance task performance. Perceived ease of use denotes the degree to which a user believes that using a given technology will be free of effort (Abdullah et al., 2016; Chen et al., 2025). In the context of GenAI, perceived usefulness mainly reflects its capacity to assist in mastering theoretical knowledge, building cognitive frameworks, filtering relevant information, facilitating academic learning, supporting foreign-language reading, aiding programming, and fostering interdisciplinary understanding (Liu and Zhang, 2025). Perceived ease of use encompasses aspects such as ease of access (e.g., no registration or payment requirements), intuitive operation, high response speed, accuracy and comprehensiveness of output, multimodal support, adaptability, and privacy protection.

Accordingly, this study proposes the following hypotheses:

*H1*: The perceived usefulness of GenAI tools has a positive effect on university students’ behavioral intentions to use them.

*H2*: The perceived ease of use of GenAI tools has a positive effect on university students’ behavioral intentions to use them.

Following the theoretical logic of TAM and TPB, behavioral intention serves as a direct antecedent of actual usage behavior. Individuals with stronger behavioral intentions are more likely to translate these intentions into real-world actions. Thus, the following hypothesis is proposed:

*H3*: University students’ behavioral intention has a positive effect on their actual usage behavior of GenAI tools.

Furthermore, social norms often exert a significant influence on technology adoption decisions. Peer and institutional expectations have been shown to shape individuals’ behavioral intentions to integrate AI into their learning processes (Kostić-Ljubisavljević and Samčović, 2024; Al-Mamary et al., 2024). Based on this, the following hypothesis is proposed:

*H4*: Subjective norms positively influence university students’ behavioral intentions to use GenAI tools.

#### 2.1.2 Perceived risk theory (PRT)

The Technology Resistance Theory posits that technology adoption decisions depend not only on perceived benefits but also on perceived risks. The PRT, introduced by Bauer at Harvard University in 1960, was initially applied to market research to explain phenomena such as information search, brand loyalty, reference groups, and purchasing decisions (Bettman, 1973). Its core idea is that consumers’ decision-making is accompanied by subjective perceptions of uncertainty regarding outcomes, and perceived risk consists of both uncertainty and consequence severity (Barach, 1969). In the context of GenAI, perceived risk can be divided into three categories:

(1) Perceived Personal Risk (PPR).

When individuals begin using new technologies, they often encounter personal concerns that may hinder adoption. This evaluation process involves critical thinking (Walter, 2024), whereby users assess information produced by GenAI tools such as ChatGPT or Midjourney before forming judgments and making decisions (Shi et al., 2020; Li, 2025; Huang et al., 2025). Students may perceive personal academic risks such as exposure to misleading information, reduced critical or creative thinking, weakened expression and analytical ability, or the use of inaccurate or fabricated data (Zhang L. et al., 2025; Zhang X. et al., 2025; Huang and Wu, 2025; Chan and Lee, 2025).

(2) Perceived Academic Environmental Risk (PER).

Beyond individual concerns, academic environmental risks also affect adoption intentions. Although GenAI technologies have transformed academic practices, they simultaneously pose challenges, such as undermining academic integrity and reducing the effectiveness of plagiarism detection systems (Huang et al., 2025). Zhou’s natural and longitudinal experimental studies demonstrated that using ChatGPT may diminish sustained creative output and lead to content homogenization (Zhou et al., 2025).

(3) Perceived Social Risk (PSR).

Featherman and Pavlou (2003) argued that higher perceived social risks related to a product or service may reduce users’ willingness to recommend it, particularly under peer influence. In the case of GenAI, social risks include concerns over information authenticity, employment displacement, academic misconduct, and technological advancement surpassing regulatory oversight (Hamed et al., 2024; Salari et al., 2025).

Accordingly, the following hypotheses are proposed:

*H5*: Perceived personal academic risk negatively affects university students’ behavioral intentions to use GenAI tools.

*H6*: Perceived academic environmental risk negatively affects university students’ behavioral intentions to use GenAI tools.

*H7*: Perceived social risk negatively affects university students’ behavioral intentions to use GenAI tools.

#### 2.1.3 Knowledge attitude practice (KAP)

The Knowledge–Attitude–Practice model is one of the most widely used frameworks to explain how individual knowledge and beliefs influence behavioral change, particularly in the context of health behavior research. The model suggests that knowledge serves as the precursor to attitude formation, and attitudes, in turn, drive behavioral practice (Andrade et al., 2020; Salama et al., 2025). Knowledge reflects awareness and understanding of relevant information; attitude represents an individual’s positive or negative evaluation toward an object; and practice denotes the habitual or intentional behaviors exhibited in real-world contexts (Zhang et al., 2023). Typically, individuals acquire new knowledge that shapes their attitudes, which subsequently influence their behavioral actions (Ritter et al., 2025).

In the context of GenAI, this study examines university students’ levels of cognitive understanding—such as their awareness of GenAI concepts, familiarity with machine learning principles, understanding of AI “hallucination” phenomena, and attitudes toward AI-generated content—to assess how these knowledge components influence their behavioral intentions. Accordingly, the following hypothesis is proposed:

*H8*: University students’ technology awareness of GenAI positively influences their behavioral intentions to use them.

### 2.2 Explainable machine learning

#### 2.2.1 Implementation of machine learning

The machine learning process consists of three key steps: feature input, model construction, and performance evaluation (Qian et al., 2024; Qiu et al., 2025). We selected input variables that are likely to have a significant impact on students’ use of GenAI tools. Specifically, these include measurement variables under the latent constructs that significantly influence the students’ behavioral intentions path in the structural equation model. During the model construction phase, we employed eight machine learning algorithms: Linear Regression, Lasso Regression, Ridge Regression, K-Nearest Neighbors, Support Vector Regression, Random Forest Regression, XGBoost, and Gradient Boosting Regression.

To ensure optimal performance, we followed rigorous training protocols in applying machine learning algorithms. Hyperparameter tuning is a critical step in achieving optimal model performance. We used a grid search strategy to identify the best combination of parameters and fine-tuned them using cross-validation. Specifically, the dataset was randomly partitioned into k folds for k -fold cross-validation, where each iteration trained the model on k−1 folds and validated it on the remaining fold. We set k to 10, used root mean square error (RMSE) as the optimization metric (Reid et al., 2015; Van Den Hoogen et al., 2019; Qiu et al., 2025), and reported the coefficient of determination (R^2^) to indicate the model’s goodness-of-fit between predicted and actual values. All machine learning models were implemented using Python (version 3.13).

#### 2.2.2 Explanation of machine learning models

Interpretable Machine Learning techniques were employed to enhance the transparency, credibility, and explanatory power of the predictive models. These methods facilitate a deeper understanding of the model’s internal logic, support debugging and optimization processes, and help identify key variables that most strongly influence students’ behavioral intentions toward GenAI adoption (Linardatos et al., 2020).

Two complementary IML approaches were applied to assess feature importance. The first method was based on node-splitting analysis derived from tree-based ensemble models. Specifically, the built-in get_booster() and get_score() functions in the XGBoost package (Python) were used to quantify feature importance according to the frequency and gain associated with each variable’s contribution to model improvement. This method provides a global view of variable importance within the decision tree structure. The second method employed SHAP (SHapley Additive exPlanations), a game-theoretic approach that decomposes model predictions into additive feature contributions (Jia et al., 2024; Qian et al., 2024). Using the Explainer module from the SHAP package in Python, we computed both global and local interpretations. Globally, SHAP values rank the overall importance of input features; locally, they quantify each feature’s marginal contribution to an individual prediction. A positive SHAP value indicates that a feature positively contributes to the likelihood of GenAI usage, whereas a negative value implies an inhibiting effect. By aggregating SHAP values across samples, this approach enables robust importance ranking and reveals potential interaction effects between features. A variable is considered a core influencing factor only when it is identified by two different interpretive methods simultaneously.

Collectively, these interpretable modeling strategies constitute the second stage of the proposed two-step SEM–XML framework. Following the SEM-based hypothesis testing and path analysis in the first stage, the IML-based importance analysis provides a data-driven validation and refinement of the conceptual model. This systematic integration of path analysis and factors identification strengthens the methodological robustness of the study and yields deeper insights into the behavioral mechanisms underlying GenAI adoption in higher education.

### 2.3 Summary of conceptual model and research framework

This section synthesizes the conceptual model and 2SSX research framework of the study, presenting an integrated overview of the theoretical constructs and analytical procedures that guide the research process. Building upon the extended TAM, the TPB, the PRT, and the KAP framework discussed earlier, this section consolidates the hypothesized relationships and methodological design into a unified view that includes a theory driven conceptual model and a two step analytical workflow.

**Figure 1:**
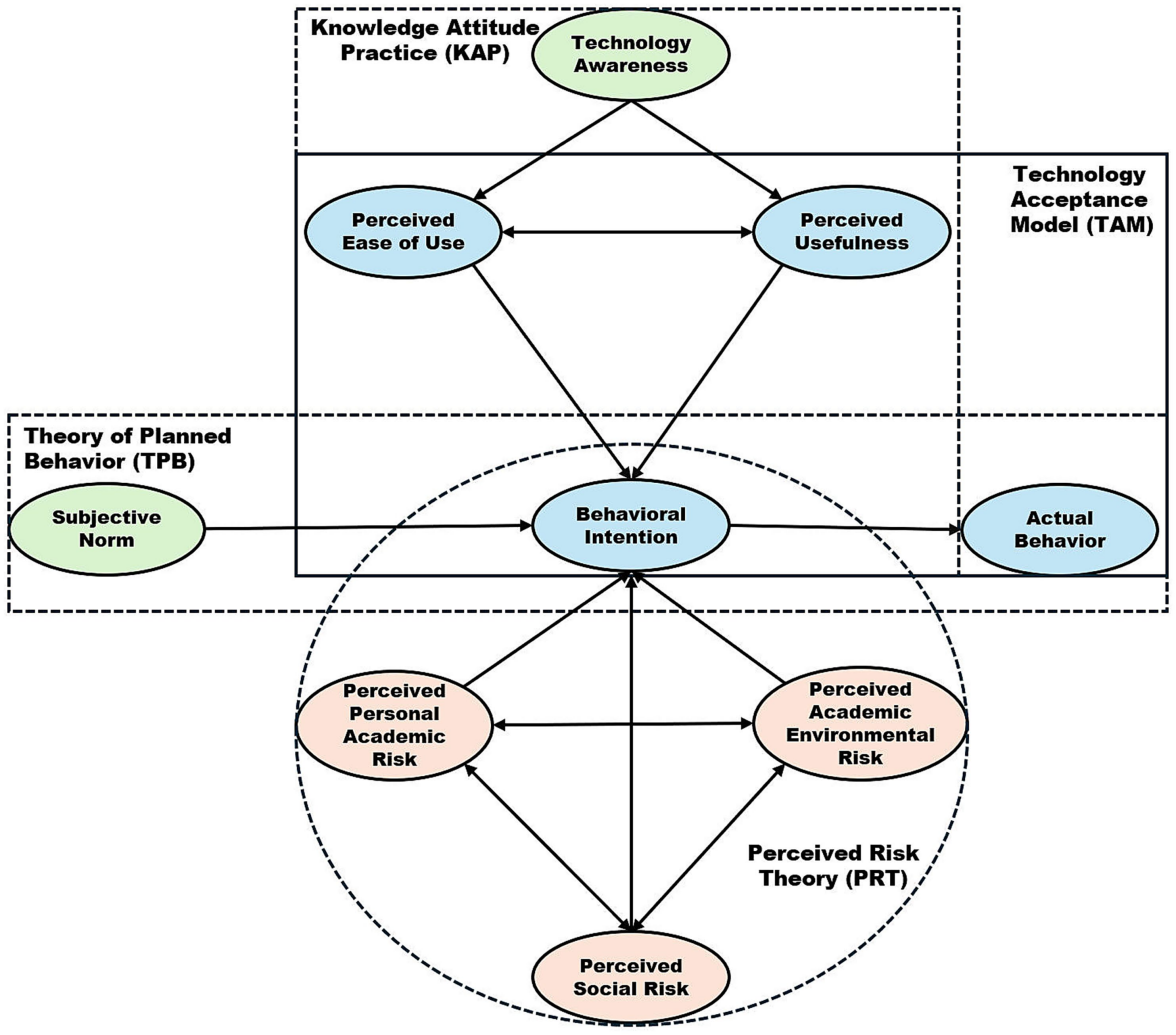
Conceptual model.

Figure 1 illustrates the conceptual model of the study, which outlines the hypothesized determinants influencing university students’ intentions to adopt GenAI tools. The model integrates constructs related to both benefits and risks. On the positive side, perceptions such as perceived usefulness, ease of use, subjective norms, and knowledge of GenAI are hypothesized to increase students’ behavioral intentions to use these tools. Conversely, negative perceptions, such as perceived academic risks, environmental risks, and social risks, are hypothesized to inhibit such intentions. Together, these relationships form a integrated framework that captures both the motivational and inhibitory factors that shape students’ adoption behavior.

**Figure 2:**
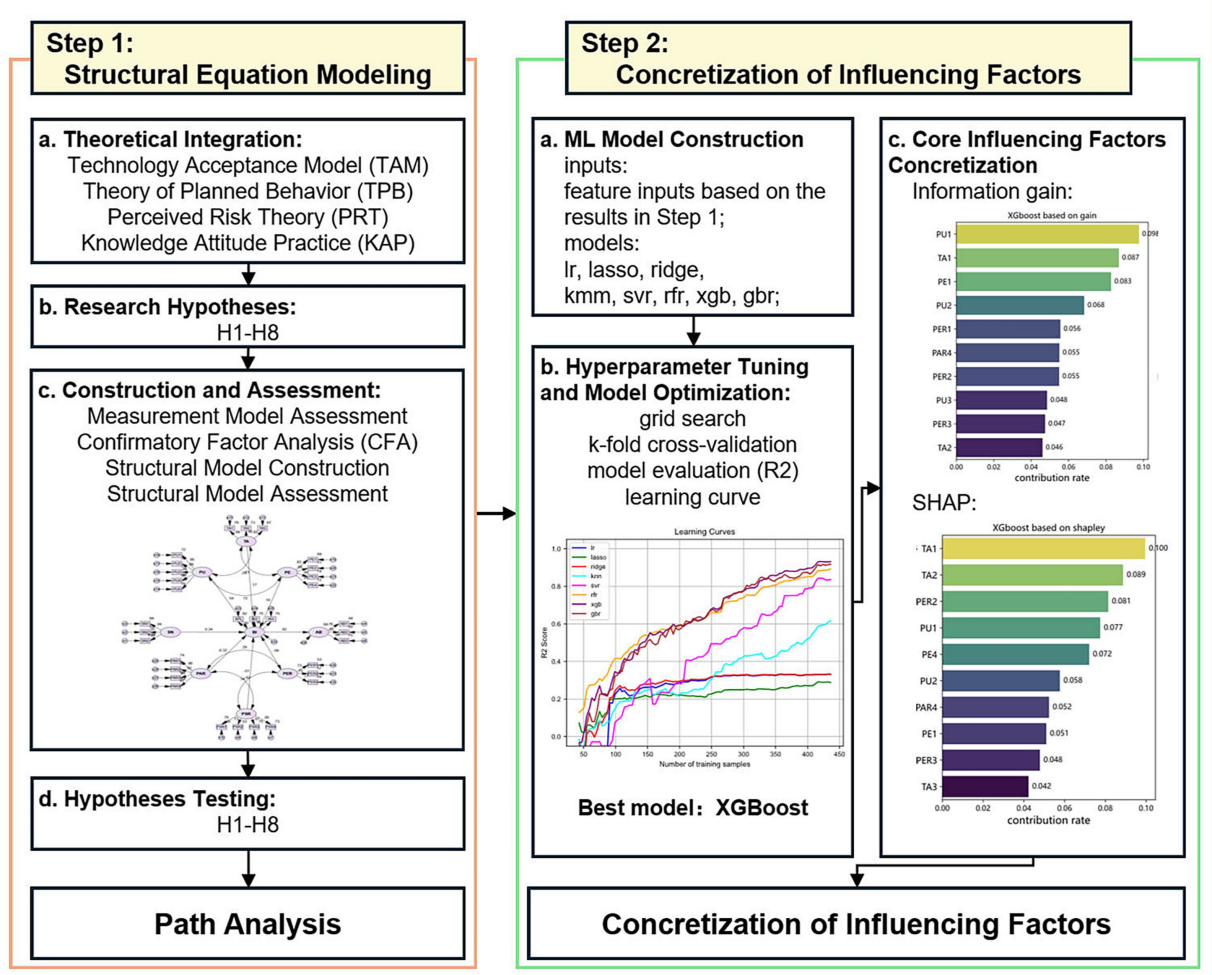
Two-step SEM–XML research framework.

Figure 2 presents the research framework of the study, which follows the 2SSX approach. This framework combines the strengths of SEM with XML, providing a two step approach that connects construct level mechanisms with indicator level factor specification to understanding GenAI adoption. The analysis is carried out in two distinct phases:

- Latent-construct mechanism modeling (SEM):

In Step 1, we employ SEM as a theory consistent mechanism model to evaluate how key latent constructs jointly shape GenAI adoption. This stage comprises (i) measurement model assessment, such as confirmatory factor analysis and reliability/validity evaluation, and (ii) structural model estimation to test the hypothesized paths H1–H8. By operating at the construct level, SEM clarifies the conceptual relationships among motivations, attitudes, perceived risks, and adoption intention. It also provides a coherent account of the underlying mechanism implied by the study’s theoretical framework.

- Step 2: Indicator-level factor concretization (XML):

In Step 2, we introduce XML to concretize the core influencing factors at the indicator level. Whereas SEM primarily identifies whether construct-level relations are supported, XML is used to characterize how specific observed indicators account for variation in adoption outcomes in the empirical data. This stage involves model construction and optimization under a consistent feature set derived from the SEM measurement system, followed by interpretable attribution/importance analyses to identify salient indicators. In this sense, XML does not function as an objective validation of SEM. Instead, it serves as an item level refinement layer that translates construct level mechanisms into empirically prominent and actionable measured factors. The XML results are therefore interpreted as indicator level salience patterns rather than causal confirmation.

- 2SSX framework:

Together, SEM and XML constitute the 2SSX framework, which links mechanism explanation at the latent construct level to factor concretization at the observable indicator level. The two steps are coupled. SEM establishes the theoretically grounded construct level mechanism, while XML highlights which measured indicators most strongly represent those constructs and are most empirically prominent for adoption related outcomes. Because Step 2 is informed by the SEM measurement structure, it should not be interpreted as an independent corroboration of SEM path significance. Moreover, the shift from latent constructs to observed indicators reduces explicit accounting for measurement error and model uncertainty. Accordingly, XML results are interpreted as complementary evidence that sharpens the understanding of “what specifically matters” at the indicator level, rather than as a direct extension of SEM causal claims.

In practical terms, this indicator-level refinement is useful when SEM identifies a significant construct-level effect but does not specify which component items are more decisive. For example, if SEM reveals that Perceived Personal Academic Risk (PAR) exerts a significant negative influence on GenAI adoption, XML can further differentiate which PAR indicators, such as weakening critical thinking (PAR1), academic originality crisis (PAR2), decline in expression and analysis abilities (PAR3), or risk of false data (PAR4), contribute more prominently to the observed adoption outcomes. Such item-level evidence enables the study to move from a general construct inference to more targeted and actionable recommendations, such as prioritizing interventions that address the most salient academic risk components, thereby improving the practical specificity of governance and educational guidance. In addition, to maintain conceptual and analytical consistency across the two steps, construct relevance identified in Step 1 is used to guide feature specification in Step 2. For instance, if subjective norm (SN) shows a non-significant association with GenAI adoption in the SEM results, the corresponding subjective norm indicators (SN1, SN2, SN3) are excluded from the XML feature set so that the indicator level analysis remains aligned with the construct level mechanism supported by SEM.

Although SEM models measurement error at the latent construct level, the indicator level XML analysis does not explicitly propagate the same measurement error structure or uncertainty into the second step. This methodological gap is acknowledged, and the XML findings are used to specify salient indicators and practical priorities rather than to draw causal conclusions.

## 3 Questionnaire design and data collection

### 3.1 Questionnaire design

The questionnaire was designed from three main dimensions. The first section collected demographic information; the second focused on GenAI usage patterns, including frequency, motivations, purposes, and preferences; and the third explored behavioral intentions and actual usage drivers, covering students’ perceptions of GenAI’s convenience, accuracy, diversity, timeliness, and privacy protection. It also examined the perceived positive or negative academic impacts, potential issues such as information authenticity, cognitive weakening, legal or ethical violations, and privacy concerns, as well as respondents’ views on GenAI’s technological innovation and development trends. To ensure content validity and reduce potential misunderstanding of questionnaire items, a pilot test was conducted with 15 participants. Feedback from the pilot helped refine the wording and structure of the items.

The final version of the questionnaire consisted of three demographic questions (gender, academic level, and major) and nine multi-item constructs measured on a five-point Likert scale (1 = “strongly disagree” to 5 = “strongly agree”). These constructs were: Technology Awareness (TA), Perceived Ease of Use (PE), Perceived Usefulness (PU), Perceived Personal Academic Risk (PAR), Perceived Academic Environmental Risk (PER), Perceived Social Risk (PSR), Subjective Norm (SN), Behavioral Intention (BI), and Actual Behavior (AB). Each construct was measured using several reflective indicators derived from established scales in prior literature, adapted to the context of GenAI use in higher education.

### 3.2 Data collection

This study aims to investigate the factors influencing undergraduate students’ acceptance of GenAI tools as regular academic aids and to provide a multidimensional assessment of their usage cognition. By systematically analyzing the collected data, the research seeks to reveal how GenAI affects students’ learning processes and offer practical insights for its application in educational settings.

A quantitative survey method was employed. The questionnaire was distributed to undergraduate, master’s, and doctoral students in several universities across Southwestern China. A two-stage sampling method was adopted. In the first stage, universities of different types (comprehensive, technological, and normal universities) were selected. In the second stage, participants were randomly sampled across various disciplines and academic levels.

Data collection took place from March to April 2025. Before the formal survey, 100 online questionnaires were distributed to students in Southwest China. Based on the pre-survey data, the average sample standard deviation (s) was calculated as 0.746. The absolute margin of error (d) for each question was set to 0.05, and with a 95% confidence level. The sample size calculation formula is as follows (Singh and Masuku, 2014)


(1) $$n={\left(\frac{z_{1-\alpha /2}s}{d}\right)}^{2}$$

Where $z_{1-\alpha /2}$ is the upper percentile of the normal distribution, s is the sample standard deviation for each questionnaire question, n is the number of respondents for each questionnaire question, and d is the pre-set absolute margin of error.

The sample size was determined to be 856, calculated using Equation 1. Considering potential issues such as invalid questionnaires, the final sample size was set to 900, and therefore, an additional 800 questionnaires were collected based on the pre-survey. In total, 900 questionnaires were distributed—420 offline and 480 online—covering students from diverse academic backgrounds, including science and engineering, humanities, and social sciences. After excluding incomplete responses, patterned answers, extremely short completion times, and outliers, 880 valid questionnaires were retained, yielding an effective response rate of 97.78%.

### 3.3 Descriptive analysis of the sample

As shown in Table 1, among the valid responses, 51.58% of participants were male and 48.42% were female. Regarding academic level, undergraduates comprised 66.47% of the sample, including 45.11% from first- and second-year students and 21.36% from third- and fourth-year students. Graduate students accounted for 20.79%, while associate degree and doctoral students represented 7.16 and 5.57%, respectively. In terms of disciplinary distribution, students majoring in engineering (20.11%), economics (19.55%), and science (16.70%) formed the largest groups, together accounting for 56.36% of all respondents. Respondents from philosophy, arts, and interdisciplinary fields were relatively few.

**Table 1:** Profile of the interviewees (*n* = 880).

Items	Values	Frequency	Percentage (%)
Gender	Male	427	48.52
	Female	453	51.48
Grade	Junior college	63	7.16
	Undergraduate 1st–2nd year	397	45.11
	Undergraduate 3rd–4th year	188	21.36
	Master 1st year	94	10.68
	Master 2nd–3rd year	89	10.11
	Doctor	49	5.57

Major	Percentage (%)
Engineering	20.11
Economics	19.55
Science	16.70
Management	14.77
Education	6.70
Literature	4.66
Medicine	4.09
Law	3.52
History	3.30
Agriculture	2.84
Art	1.48
Philosophy	1.14
Interdisciplinary	1.14
Military Science	0.00

GenAI tools have achieved a high penetration rate among university students. Among the respondents, 78% of university students have used GenAI tools, while only 22% have not. Of the students who have not used these tools, 34.5% expressed a willingness to use GenAI tools in the future. University students mainly use GenAI tools for master theoretical knowledge (e.g., answering individual concepts or basic issues) (79.21%), building a knowledge framework (e.g., understanding the distinctions and logical connections between related concepts) (59.74%), and quickly grasp interdisciplinary knowledge (55.20%). Overall, their usage focuses on basic functions rather than advanced features like providing ideas or data mining, reflecting a surface-level engagement with the tools.

Besides, the variable codes and their corresponding meanings are shown in Table 2. In the following analysis, these codes will be used to refer to the variables associated with each item.

**Table 2:** Main variables.

Variable	Explanation
TA	Technology awareness
TA1	Understand the concepts of AI, AGI, and GenAI, and distinguish between them.
TA2	Have a basic understanding of the principles behind machine learning, deep learning, and related technologies.
TA3	Be aware of the “hallucination” phenomenon in GenAI, including its causes and implications.
PE	Perceived Ease of Use
PE1	Easy to operate and learn.
PE2	Accurately identifies user intentions.
PE3	High answer accuracy.
PE4	Outputs comprehensive content.
PU	Perceived Usefulness
PU1	Master theoretical knowledge by providing clear explanations.
PU2	Building a comprehensive knowledge framework.
PU3	Mastering interdisciplinary knowledge.
PU4	Assisting with reading foreign literature.
PU5	Mastering typical analysis methods.
PAR	Perceived Personal Academic Risk
PAR1	Weakening critical thinking.
PAR2	Academic originality crisis.
PAR3	Decline in expression and analysis abilities.
PAR4	Risk of false data.
PER	Perceived Academic Environmental Risk
PER1	Dilute academic integrity.
PER2	Plagiarism check systems to fail.
PER3	Duplication of academic results.
PSR	Perceived Social Risk
PSR1	Information authenticity risk.
PSR2	Employment impact risk.
PSR3	Academic misconduct risk.
PSR4	Development outpacing human regulation ability.
SN	Subjective Norm
SN1	Suggestions from classmates, friends, or teachers.
SN2	Behaviors of classmates, friends, or teachers.
SN3	National policies and school regulations.
BI	Behavior Intention
BI1	Be willing to use GenAI tools more reasonably in the future.
BI2	Be willing to use new/better GenAI tools in the future.
BI3	Be willing to recommend GenAI tools to classmates or friends in the future.
AB	Actual Behavior
AB1	Priority level for using GenAI tools.
AB2	Frequency of using GenAI tools.
AB3	Recommend friends or classmates to use GenAI tools.

All variables are included in SEM, while the Perceived Social Risk (PSR) variables that have an insignificant effect on Behavior Intention (BI) are excluded from the XML model.

### 3.4 Group differences in perceived benefits and risks

Different groups exhibit distinct characteristics. We applied binary 01 coding to the “usage” variable (whether used or not) and ordinal coding to the “grade” variable, grouping the data accordingly. The group means, correlation coefficients, and analysis of ANOVA were calculated for their relationships with perceived benefits and perceived risks. Levene’s Test was used to check for homogeneity of variance (null hypothesis: equal variances). If variance was homogeneous, standard ANOVA was applied; otherwise, Welch’s ANOVA was used. The results are presented in Table 3. As shown in Table 3, significant inter-group differences were found based on GenAI tool usage. The group that used GenAI tools reported significantly higher perceived benefits and perceived risks. Significant differences were also found across groups of undergraduates (including associate degree students), graduate students, and PhD students. The relationship with education level was non-linear, with master’s students reporting the highest perceived benefits and risks, while PhD students had significantly lower perceived benefits than both undergraduates and master’s students.

**Table 3:** Group differences.

	Usage		Grade		
	Have used	Have not used	Undergraduate	Master	PhD
Observations	683	197	648	183	49
Perceived benefits					
Mean	3.649	3.221	3.53	3.776	3.02
Pearson correlation coefficient	Pearson = 0.216***		Pearson = −0.027		
Levene’s Test	Levene = 0.128		Levene = 29.553***		
ANOVA	*F* = 42.890***		*F* = 11.745***		
Perceived risk					
Mean	3.697	3.161	3.553	3.675	3.526
Pearson correlation coefficient	Pearson = 0.381***		Pearson = 0.044		
Levene’s Test	Levene = 4.700**		Levene = 2.722*		
ANOVA	Welch_F = 117.716***		*F* = 3.288**		

Stars indicate statistical significance, with *p < 0.05, **p < 0.01, and ***p < 0.001.

## 4 Results and discussion

### 4.1 Path analysis (step 1)

#### 4.1.1 Measurement model assessment

According to established methodological standards (Albayati, 2024; Leguina, 2015; Diamantopoulos and Siguaw, 2000; Yu et al., 2025), it is necessary to evaluate the reliability, validity, factor loadings, convergent validity, composite reliability and discriminant validity of all measurement items.

Based on prior literature, the reliability and construct validity of each measurement item were first examined. Reliability reflects the internal consistency of the measurement results—if an instrument produces stable results under the same conditions, it is considered reliable. Higher reliability indicates stronger inter-item correlations, implying that the items measure the same latent construct consistently. Reliability is typically assessed using Cronbach’s *α*, with a threshold value above 0.70 indicating acceptable reliability. Construct validity determines whether the measurement instrument accurately captures the theoretical construct which intends to measure. It reflects the correspondence between the observed indicators and the underlying latent variables. Higher construct validity implies that the items can effectively differentiate between distinct latent constructs, making the data suitable for factor analysis. The Kaiser–Meyer–Olkin (KMO) measure of sampling adequacy is commonly used, with a minimum acceptable value of 0.60 for each construct.

To further verify the absence of common method bias, a Harman Single-Factor Test was conducted. The test results showed that the first principal component explained 34.2% of the total variance, which is below the 40% threshold, indicating that common method bias is not a concern in this study.

As shown in Table 4, all constructs exhibit strong reliability and validity. Specifically, all Cronbach’s *α* values exceed 0.70; except for Perceived Academic Environmental Risk (0.795) and Subjective Norm (0.727), all others exceed 0.85, indicating robust internal consistency. Furthermore, all KMO values are greater than 0.70, confirming the suitability of the data for factor analysis and indicating that the items effectively represent their respective constructs. The square root of the AVE for each latent variable is greater than the standardized correlation coefficients outside the diagonal (see Table 5), which further supports the good discriminant validity of the latent variables.

**Table 4:** Reliability and validity testing results.

Constructs	Cronbach’s α	KMO
Technology awareness (TA)	0.891	0.745
Perceived ease of use (PEU)	0.919	0.855
Perceived usefulness (PU)	0.934	0.904
Perceived personal academic risk (PAR)	0.881	0.810
Perceived academic environmental risk (PER)	0.795	0.729
Perceived social risk (PSR)	0.867	0.708
Subjective norm (SN)	0.727	0.812
Behavioral intention (BI)	0.914	0.759
Actual behavior (AB)	0.890	0.731

KMO (Kaiser–Meyer–Olkin) measure of sampling adequacy assesses the suitability of data for factor analysis.

**Table 5:** Discriminant validity testing results.

	TA	PE	PU	PAR	PER	PSR	SN	BI	AB
TA	0.856								
PE	0.494	0.862							
PU	0.530	0.672	0.860						
PAR	−0.232	−0.319	−0.352	0.814					
PER	−0.407	−0.184	−0.193	0.446	0.752				
PSR	−0.225	−0.486	0.273	0.159	0.296	0.791			
SN	0.212	0.125	0.114	−0.142	−0.084	−0.261	0.865		
BI	0.257	0.194	0.609	−0.274	−0.107	−0.055	0.378	0.883	
AB	0.323	0.325	0.535	−0.307	−0.284	−0.113	0.215	0.722	0.822

The diagonal represents the square root of the AVE (Average Variance Extracted), while the values below the diagonal represent the correlation coefficients between the variables. AVE represents the mean variance captured by a construct’s indicators, with values greater than 0.5 suggesting that the construct explains more than half of the variance in its indicators.

#### 4.1.2 Confirmatory factor analysis

To further assess whether observed variables effectively measure the latent constructs, Confirmatory Factor Analysis (CFA) was conducted. The model’s quality was evaluated using factor loadings, Squared Multiple Correlations (SMC), Composite Reliability (CR) and Average Variance Extracted (AVE) (Lu et al., 2025). (1) Factor loading represents the standardized correlation between an observed variable and its corresponding latent construct. (2) SMC indicates the proportion of variance in an observed variable explained by the latent factor. (3) Convergent validity measures the degree of agreement among items assessing the same construct, typically assessed by AVE, where a value above 0.50 indicates satisfactory convergence. (4) CR provides a more accurate estimate of internal consistency than Cronbach’s α during CFA, with values above 0.70 considered acceptable.

Measurement items with factor loadings below 0.70 or SMC values below 0.50 were removed and renumbered. The revised indicators are presented in Table 6. All constructs achieved CR > 0.70 and AVE > 0.50, confirming adequate convergent validity and internal consistency reliability.

**Table 6:** Confirmatory factor analysis results.

Latent variable	Indicators	Loading	SMC	AVE	CR
		>0.70	>0.50	>0.50	>0.70
Technology awareness (TA)	TA1	0.893	0.797	0.732	0.891
	TA2	0.847	0.717		
	TA3	0.825	0.681		
Perceived ease of use (PE)	PE1	0.852	0.726	0.743	0.935
	PE2	0.892	0.796		
	PE3	0.894	0.799		
	PE4	0.804	0.646		
	PE5	0.865	0.748		
Perceived usefulness (PU)	PU1	0.819	0.671	0.740	0.919
	PU2	0.888	0.789		
	PU3	0.880	0.774		
	PU4	0.851	0.724		
Perceived personal academic risk (PAR)	PAR1	0.865	0.748	0.662	0.885
	PAR2	0.916	0.839		
	PAR3	0.812	0.659		
	PAR4	0.734	0.539		
Perceived academic environmental risk (PER)	PER1	0.746	0.557	0.565	0.796
	PER2	0.780	0.608		
	PER3	0.728	0.530		
Perceived social risk (PSR)	PSR1	0.702	0.493	0.626	0.869
	PSR2	0.726	0.527		
	PSR3	0.853	0.728		
	PSR4	0.872	0.760		
Subjective norm (SN)	SN1	0.789	0.623	0.748	0.899
	SN2	0.864	0.747		
	SN3	0.936	0.876		
Behavioral intention (BI)	BI1	0.891	0.794	0.779	0.914
	BI2	0.868	0.753		
	BI 3	0.889	0.790		
Actual behavior (AB)	XW1	0.769	0.591	0.676	0.862
	XW2	0.841	0.707		
	XW3	0.854	0.729		

SMC (Squared Multiple Correlation) represents the proportion of variance in an observed variable explained by its underlying factors, indicating how well the factors explain the variability in the variable. CR (Composite Reliability) measures the internal consistency or reliability of a latent construct.

#### 4.1.3 Structural model assessment

**Figure 3:**
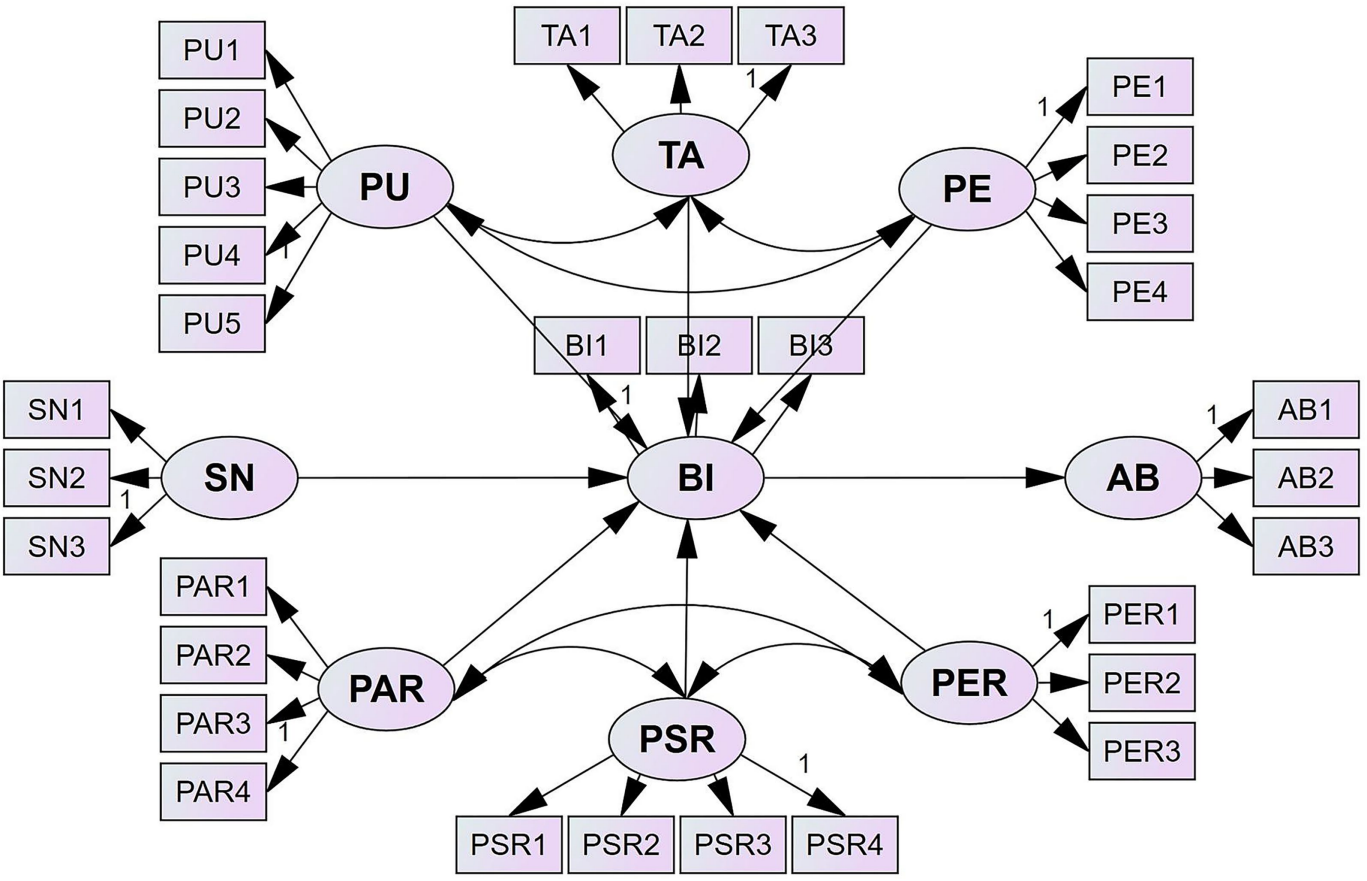
Structural model results.

Drawing from the theoretical framework and the results of the CFA, a Structural Equation Model (SEM) was constructed using AMOS 26.0 for model estimation. Figure 3 illustrates the SEM structure and standardized path coefficients, while Table 7 presents the model fit indices.

**Table 7:** Structural model assessment results.

Indexes	Estimates	Results
χ/df	2.863	Good
RMSEA	0.043	Perfect
CFI	0.923	Good
IFI	0.954	Perfect
TLI	0.895	Acceptable
NFI	0.933	Good

Model-data fit was evaluated by examining fit indices. RMSEA (Root Mean Square Error of Approximation) measures how well the model fits the population covariance matrix. CFI (Comparative Fit Index) compares the fit of the hypothesized model to a baseline model. IFI (Incremental Fit Index) also evaluates model fit by comparing the hypothesized model to a baseline. TLI (Tucker–Lewis Index) assesses model fit by comparing the chi-square value of the model to a null model. NFI (Normed Fit Index) compares the chi-square value of the proposed model to a baseline. The obtained fit indices were compared with criterion values to interpret model-data fit. The criteria were as follows: $\begin{array}{l} \chi /\mathrm{df}\leq 2.00,\text{RMSEA}\leq 0.05,\mathrm{CFI}\geq 0.95,\mathrm{IFI}\\ \geq 0.95,\mathrm{TLI}\geq 0.95,\mathrm{NFI}\geq 0.95\; \end{array}$ indicated a perfect fit; $\begin{array}{l} \chi /\mathrm{df}\leq 3.00,\text{RMSEA}\leq 0.08,\mathrm{CFI}\geq 0.90,\mathrm{IFI}\\ \geq 0.90,\mathrm{TLI}\geq 0.90,\mathrm{NFI}\geq 0.90\; \end{array}$ indicated a good fit; $\begin{array}{l} \chi /\mathrm{df}\leq 5.00,\text{RMSEA}\leq 0.08,\mathrm{CFI}\geq 0.85,\mathrm{IFI}\\ \geq 0.85,\mathrm{TLI}\geq 0.85,\mathrm{NFI}\geq 0.85\; \end{array}$ indicated an acceptable/moderate fit (Tabachnick et al., 2007; Brown, 2015; Kline, 2023; Delibalta et al., 2025; Hu and Bentler, 1999). In Table 7, all model fit indices meet or exceed recommended standards, except for the slightly lower TLI, indicating overall satisfactory model fit ( χ /df = 2.863, RMSEA = 0.043, CFI = 0.923, IFI = 0.954, TLI = 0.895, NFI = 0.933). These results suggest that the model is suitable for explaining both the relationships between latent and observed variables and the structural paths among latent constructs.

#### 4.1.4 Hypotheses testing and discussion

As shown in Table 8, the results of this paper found that:

- H1. (*β* = 0.100, *p* < 0.05) The analysis reveals that Perceived Ease of Use has a significant positive effect on Behavioral Intention (*β* = 0.100, *p* < 0.05). This suggests that students who perceive GenAI as easy to use are more likely to express a stronger intention to adopt it. The ease with which students can interact with GenAI tools, both in terms of user interface and accessibility, plays a crucial role in fostering adoption. These findings align with prior research, which emphasizes the importance of usability in technology acceptance.
- H2. (*β* = 0.544, *p* < 0.001) Perceived Usefulness has an even more substantial influence on Behavioral Intention (BI) (*β* = 0.544, *p* < 0.001), demonstrating that the perceived practical value of GenAI strongly enhances students’ intention to use the technology. This result supports the notion that students are more likely to engage with GenAI when they believe it offers tangible benefits, such as improving academic performance or facilitating learning tasks. The significant path coefficient here underscores the importance of aligning GenAI tools with students’ academic needs and objectives.
- H3. (*β* = 0.824, *p* < 0.001) Behavioral Intention is found to be a strong predictor of Actual Behavior (*β* = 0.824, *p* < 0.001), representing the most robust path in the model. This indicates that students who exhibit a higher intention to use GenAI tools are more likely to engage in frequent and consistent usage. The high explanatory power of this relationship supports the theory that behavioral intention is a key determinant of actual technology adoption and usage behavior.
- H4. (*β* = 0.339, *p* < 0.001) Subjective Norm, which captures the influence of peers and institutional expectations, significantly affects Behavioral Intention (*β* = 0.339, *p* < 0.001). This result suggests that students’ intentions to adopt GenAI are shaped not only by their personal perceptions of the technology but also by external social pressures. Institutional support, peer recommendations, and the broader academic environment play an essential role in shaping adoption intentions. This finding underscores the importance of social and institutional contexts in the adoption process.
- H5. (*β* = −0.316, *p* < 0.001) Perceived Personal Academic Risk has a significant negative effect on Behavioral Intention (*β* = −0.316, *p* < 0.001). Concerns regarding the potential negative impact of GenAI on critical thinking, creativity, and academic independence significantly deter students from adopting the technology. These concerns reflect broader debates around the ethical use of AI in academic contexts and highlight the need for educational institutions to address such risks when promoting GenAI usage.
- H6. (*β* = −0.094, *p* < 0.05) Similarly, Perceived Academic Environmental Risk exerts a significant negative effect on Behavioral Intention (*β* = −0.094, *p* < 0.05). Students’ worries about issues like plagiarism detection failure and the erosion of academic originality are significant barriers to GenAI adoption. This finding highlights the need for universities and policymakers to establish clear guidelines for responsible AI use to mitigate such concerns.
- H7. (*β* = −0.008, *p* > 0.05) Interestingly, the effect of Perceived Social Risk on Behavioral Intention is not significant (*β* = −0.008, *p* > 0.05), suggesting that societal concerns about reputation or social perception do not significantly affect students’ decisions to use GenAI tools. In the context of higher education, students appear to prioritize the academic and functional of technology over external social pressures. A possible explanation is a timing effect among students in Southwest China, where social risks related to AI have not yet become a pressing concern. For these students, GenAI primarily enhances learning efficiency and assists in report writing. However, excessive reliance on AI, particularly when foundational knowledge is weak, may undermine their abilities and overall efficiency. Despite this, students generally see GenAI tools as valuable for improving their skills and, when used appropriately, can enhance both academic performance and employability. On an individual level, while students acknowledge AI’s potential to replace jobs, using GenAI provides a competitive edge in academics and the job market. Therefore, the conflicting influences of these factors may explain why Perceived Social Risk does not significantly impact students’ Behavioral Intention.
- H8. (*β* = 0.166, *p* < 0.001) Finally, Technology Awareness is found to positively influence Behavioral Intention (*β* = 0.166, *p* < 0.001). This implies that students with a higher level of technical understanding are more likely to adopt GenAI tools. As digital natives, students with greater technology awareness tend to engage more readily with innovative tools, underscoring the role of technical competence in fostering GenAI adoption.

**Table 8:** Hypotheses testing results.

Hypotheses	Paths			Std. B	S. E.	C. R.	*p*_value	Results
H1	BI	←	PE	0.100	0.053	2.024	0.043*	Accepted
H2	BI	←	PU	0.544	0.055	10.585	***	Accepted
H3	AB	←	BI	0.824	0.039	19.898	***	Accepted
H4	BI	←	SN	0.339	0.042	8.295	***	Accepted
H5	BI	←	PAR	−0.316	0.055	6.543	***	Accepted
H6	BI	←	PER	−0.094	0.038	−2.399	0.016*	Accepted
H7	BI	←	PSR	−0.008	0.048	−0.16	0.873	Rejected
H8	BI	←	TA	0.166	0.024	4.892	***	Accepted

*p < 0.05, **p < 0.01, ***p < 0.001 indicate statistically significant levels.

From the perspective of the hypothesis testing results, three key findings emerge:

(1) Perceived ease of use and perceived usefulness significantly promote behavioral intention and actual use.

Perceived ease of use and perceived usefulness both show significant positive effects on students’ behavioral intention and on their reported actual use. When tools are easy to operate and clearly help students complete or improve academic work, students are more likely to intend to use them and to incorporate them into real study and research activities. This finding is consistent with the TAM and emphasizes that both functional value and usable design jointly drive adoption in higher-education settings.

Implications: Developers and educators should prioritize enhancing both the user experience and the functional utility of GenAI tools. This can be achieved by simplifying operational procedures, offering guided tutorials, integrating discipline-specific applications, and incorporating GenAI into digital literacy training. These steps will help build user confidence and promote sustained engagement.

(2) Subjective norms and technology awareness significantly enhance adoption intention.

Subjective norms, reflecting perceived social expectations from teachers and peers, and technology awareness, representing understanding of GenAI’s capabilities, both significantly impact adoption intention. These findings suggest that students’ behavior is shaped by both social influence and technical understanding. It highlights the importance of peer endorsement and technological literacy as key drivers of technology acceptance in educational contexts.

Implications: Institutions should foster positive social influence through peer modeling, faculty demonstrations, and case-sharing activities. Simultaneously, they can improve technological literacy by organizing training workshops and integrating GenAI into the curriculum. Embedding GenAI in real academic tasks can also strengthen its perceived legitimacy and encourage sustained use in higher education.

(3) Perceived personal and academic risks inhibit adoption, while social risks are insignificant.

Both perceived personal academic risk and perceived academic environmental risk negatively influence GenAI adoption, while perceived social risk does not have a statistically significant effect. Students primarily express concerns about academic dependency, integrity violations, and institutional penalties, rather than societal judgment. This suggests that, in academic settings, students are more focused on institutional norms and academic outcomes than on social approval. These results are consistent with prior studies, which show that individual and institutional academic risks exert a stronger deterrent effect than generalized social risks.

Implications: Universities and technology providers should establish clear ethical guidelines and frameworks for academic integrity, offer training on responsible usage, and demonstrate legitimate academic applications of GenAI. Addressing students’ concerns about academic outcomes and providing clarity on institutional policies will be more effective than focusing on social approval. This approach will help build trust and encourage sustained engagement with GenAI technologies.

### 4.2 Concretization of influencing factors (step 2)

#### 4.2.1 ML model construction

To enhance the interpretability and robustness of the results, a series of supervised machine learning (ML) models were developed using Python and relevant libraries, including scikit-learn, XGBoost, and NumPy. These models were designed to concretize the relative importance of factors influencing students’ actual usage of GenAI tools, enabling a quantitative representation of abstract constructs. Specifically, eight regression algorithms were employed to ensure model diversity and comparative reliability: Linear Regression (LR), Lasso Regression (Lasso), Ridge Regression (Ridge), K-Nearest Neighbors (KNN), Support Vector Regression (SVR), Random Forest Regression (RFR), XGBoost (XGB), and Gradient Boosting Regression (GBR).

Each algorithm captures unique relationships between the independent variables (i.e., influencing factors) and the dependent variable (actual usage behavior). Linear models (LR, Lasso, Ridge) serve as parametric benchmarks for identifying linear associations and regularization effects. Nonlinear models such as KNN, SVR, and RFR handle complex, multidimensional data, while ensemble-based algorithms (XGBoost and GBR) combine multiple weak learners to achieve higher predictive accuracy and generalization. The modeling process followed standard ML procedures, including data preprocessing, feature standardization, and a 70:30 train-test split. All models were trained and validated on the same dataset to ensure comparability. Model performance was primarily assessed using the coefficient of determination (*R*^2^), along with learning curves to evaluate convergence and generalization.

In the first stage of SEM path analysis, it was found that PSR had an insufficiently significant impact on BI. As a result, the corresponding measurement variables for this latent variable were excluded from this stage. The explanatory variables inputted into the model consisted of the remaining complete latent variables—TA, PE, PU, SN, PAR, and PER—along with their corresponding measurement variables (see Table 4). The dependent variable, AB, was represented by the mean of its measured variables.

#### 4.2.2 Hyperparameter tuning and model optimization

Hyperparameter optimization is crucial for achieving optimal model performance. Grid search with k-fold cross-validation was employed to identify the best combination of parameters for each model. *R*^2^-based learning curves were generated to assess both training and validation performance.

**Figure 4:**
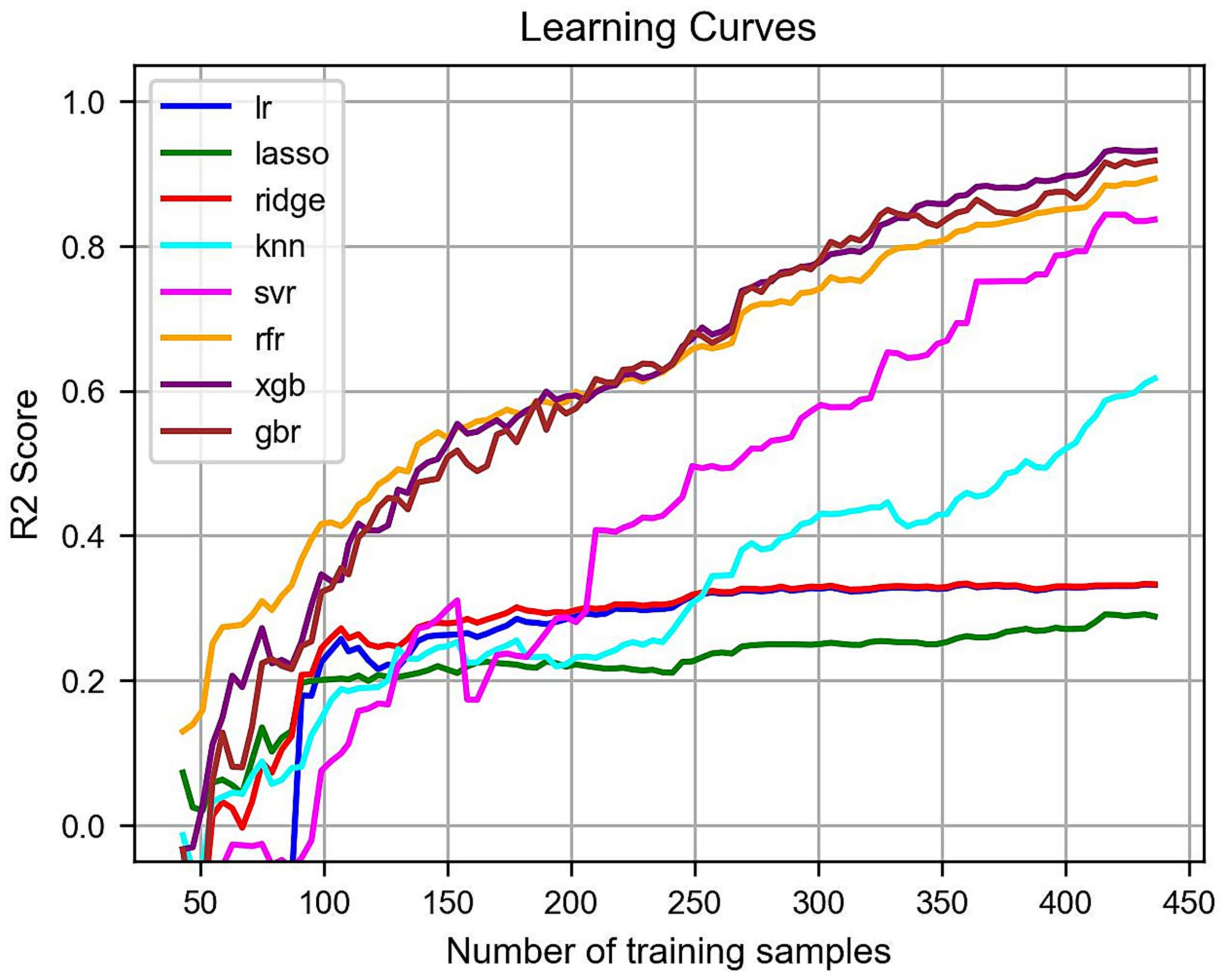
Learning curve. The learning curves of the 8 key models, which visualize the cross-validation test results during the learning and training process, with the number of training samples.

Upon evaluation, XGBoost demonstrated superior predictive accuracy and convergence (test *R*^2^ = 0.9867; cross-validation *R*^2^ = 0.9812), outperforming other algorithms (see Figure 4). Therefore, XGBoost was selected as the final model for factor concretization. Optimal hyperparameters were n_estimators = 290, learning_rate = 0.1, max_depth = 10, gamma = 0, min_child_weight = 2, subsample = 1, and colsample_bytree = 0.4. However, we acknowledge that the deep tree structure and lack of subsampling may increase the risk of overfitting. Given that the primary goal of this study is to concretize the core influencing factors, overfitting is less of a concern for the current analysis.

#### 4.2.3 Core influencing factors concretization

To further analyze the model’s prediction results, we first selected the top 10 features based on their importance scores and visualized them, as shown in Figure 4. By comparing the rankings from two different methods, we found that 8 of the top 10 features overlapped, further confirming their importance in predicting the model’s results. The average SHAP (Shapley Additive Explanations) values revealed the most influential factors in predicting students’ actual usage behavior.

**Figure 5:**
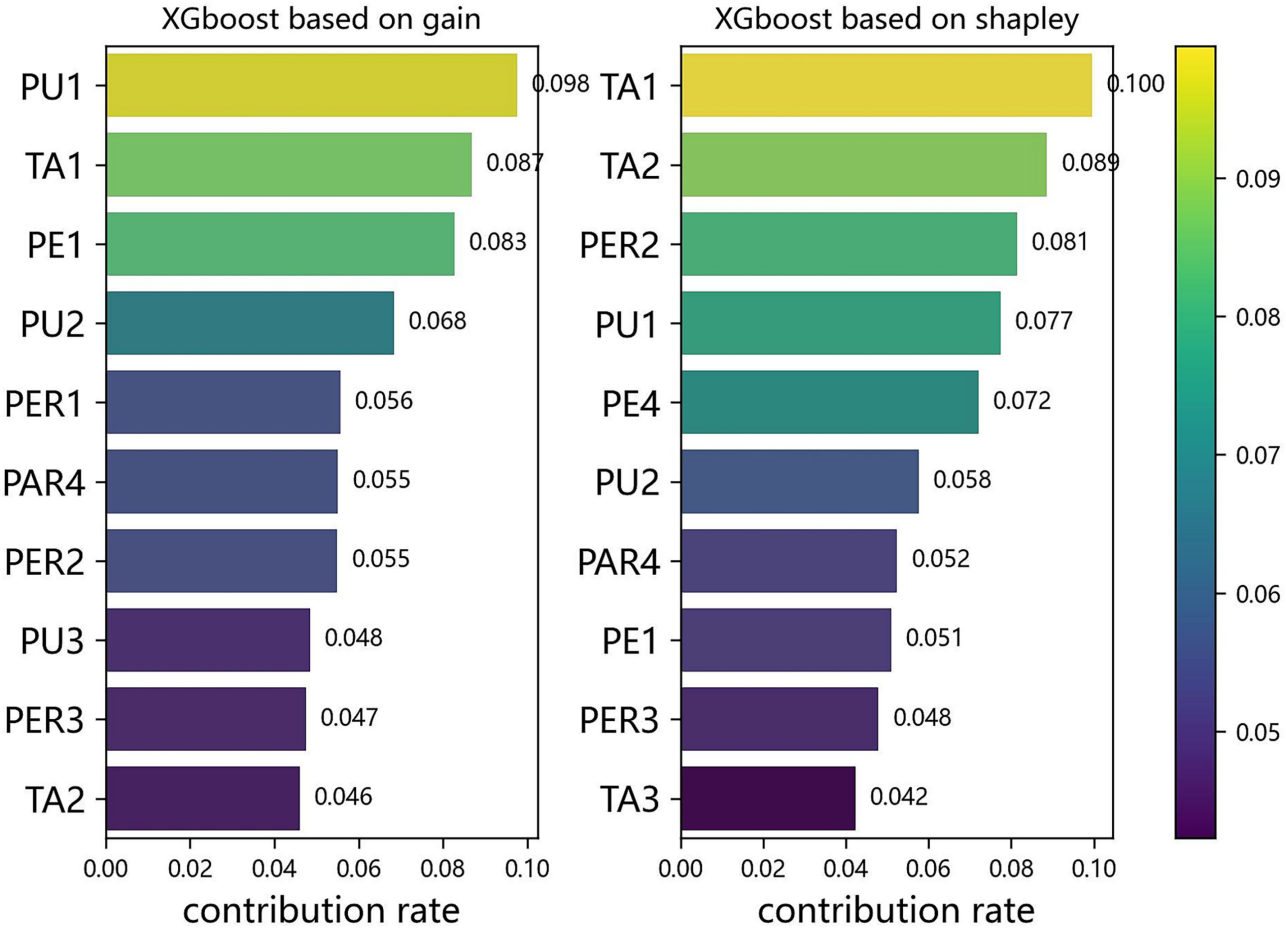
Outcomes of the concretization for core influencing factors. This visualization compares the top 10 features identified by two interpretability methods information gain (left) and SHAP.

As shown in Figure 5, TA2 (understanding of deep learning, large models, and related technical principles) ranked among the top three in both ranking methods, indicating that an understanding of technology is crucial in influencing students’ use of GenAI tools. In addition, TA1 (ability to distinguish between the concepts of AI, AGI, and GenAI) also ranked highly, highlighting that students’ basic understanding of these technologies plays a significant role in determining their use of GenAI tools. PE1 (ease of use and accessibility), PU1 (helping to master theoretical knowledge), and PU2 (supporting the establishment of knowledge frameworks) emphasized the importance of the tool’s usability and educational functionality in students’ decisions to use GenAI tools. At the same time, features such as PAR4 (risk of false data), PER2 (failure of plagiarism detection systems), and PER3 (duplication of academic results) ranked highly, reflecting students’ strong concern about academic integrity when using these tools.

Specifically, TA1 (ability to distinguish between AI, AGI, and GenAI concepts) ranked among the top three in both methods, reflecting that, in the context of AI technologies gradually permeating education and daily life, students’ ability to clearly differentiate between different types of AI impacts their willingness to adopt and effectively use these tools. Educators should focus on strengthening students’ understanding of basic AI concepts to lower the barriers to technology acceptance, build students’ confidence in using GenAI, and ultimately improve its effectiveness in actual learning. TA2 (understanding of deep learning, large models, and related technical principles) ranked highly, suggesting that students’ understanding of the fundamental principles behind deep learning and large models significantly impacts their acceptance and frequency of GenAI tool use. As generative AI technology continues to be applied in education, students’ awareness of the complexity and innovation behind these technologies becomes a key factor influencing their usage decisions.

PE1 (ease of use and accessibility), PE1 (helping to master theoretical knowledge), and PUT (supporting the establishment of knowledge frameworks) were all ranked among the top 10 in both methods, emphasizing the critical role of tool usability and perceived usefulness in students’ decisions to adopt GenAI tools. In practice, students are more likely to choose tools that are easy to use and directly aid their learning. Therefore, the usability and intuitiveness of a product will directly influence its popularity and effectiveness.

The high ranking of features like PAR4 (risk of false data), PER2 (failure of plagiarism detection systems), and PER3 (duplication of academic results) reveals students’ heightened concern about the academic credibility and integrity of generated content. In the current academic environment, issues related to the generation of false data and the failure of plagiarism detection systems have become widespread concerns. Students are increasingly worried about the authenticity and academic credibility of generated content, which may lead them to use these tools more cautiously or even limit their usage due to fears of academic misconduct.

Overall, the core factors influencing university students’ actual use of GenAI tools are not only related to technological understanding and the ease of use of the tools, but also deeply connected to multidimensional aspects such as academic integrity, data security, and learning outcomes. As GenAI technology evolves, a focus on foundational knowledge dissemination, balancing technological innovation with academic standards, ensuring usability and utility of tools, and managing potential risks will be key issues to address in future education on GenAI literacy for university students.

## 5 Conclusion, limitation, and future work

### 5.1 Conclusion

The conclusion of this study is drawn from insights into the factors influencing undergraduate students’ acceptance of GenAI tools. By adopting the novel “2SSX” research framework, the study first analyzes the risk–benefit contradiction-driven path and, in the second step, concretizes the core influencing factors. The aim of this research is to uncover the intrinsic drivers behind students’ adoption of GenAI tools and to explore the potential benefits and challenges of GenAI in educational contexts from the perspective of university students as key participants.

Grounded in the Technology Acceptance Model and the Theory of Planned Behavior, the study identifies through path analysis that internal factors, such as perceived ease of use and perceived usefulness, along with external factors like subjective norms, significantly and positively influence students’ behavioral intention to use GenAI tools. These findings are consistent with those of Albayati (2024), Zhang L. et al. (2025), and Zhang X. et al. (2025). When students perceive GenAI as easy to use, their overall attitude toward the tool becomes more positive. Similarly, when students recognize the benefits of GenAI, their intention to adopt the tool increases. Furthermore, subjective norms, as an external social influence, also have a significant positive impact on behavioral intention. When students observe their friends, classmates, or instructors using GenAI and encouraging its adoption, their willingness to use the tool is significantly enhanced.

Building on the Technology Resistance Theory and Perceived Risk Theory, the study reveals that perceived personal academic risks and perceived academic environment risks negatively affect behavioral intention, although the negative impact of perceived social risk is not significant. When students perceive potential personal academic risks associated with using GenAI tools, such as a decline in critical thinking or the risk of academic misconduct, their intention to use the tool decreases. Similarly, when students perceive academic environment risks, such as the failure of traditional plagiarism detection systems or the erosion of academic integrity, their willingness to use GenAI tools is reduced. However, students’ perception of social risks, such as job replacement concerns, does not significantly diminish their intention to adopt GenAI tools. These findings are consistent with those of Zhang and Wang (2025) and Wang et al. (2025).

According to the Knowledge-Attitude-Practice (KAP) model, the study finds that students’ level of understanding of GenAI technology significantly and positively influences their intention to use the tool. The deeper the students’ understanding of technology and their awareness of phenomena like “hallucinations” in GenAI, the stronger their intention to adopt the tool.

Using explainable machine learning methods, the study identifies key influencing factors through model construction, hyperparameter optimization, and the concretization of core factors. The findings show that TA2 (understanding of deep learning, large models, and related technical principles) is the most crucial influencing factor, significantly driving students’ intention to adopt GenAI tools. Other core factors include TA1 (ability to distinguish between AI, AGI, and GenAI concepts), PE1 (ease of use), PU1 (helps in mastering theoretical knowledge), PU2 (assists in building knowledge frameworks), and risks like PAR4 (false data risk), PER2 (failure of plagiarism detection systems), and PER3 (academic result duplication). The more students understand the tool, the more confident they are in using it, and the more willing they are to adopt new technologies. The practical value of the tool—both in its superficial (mastering theoretical knowledge) and deeper (building knowledge frameworks) functionalities—is a core driver behind students’ use of GenAI in learning and research. Moreover, students are more concerned with personal risks related to false data and academic environment risks like plagiarism detection system failures and academic result duplication. These concerns diminish their willingness to use the tools.

In conclusion, this study offers valuable insights into understanding the dynamic mechanisms behind students’ acceptance of GenAI tools. It highlights the importance of perceived ease of use, usefulness, risk perceptions, and tool understanding from the perspective of student users. By focusing on these dimensions, educators and administrators can effectively integrate GenAI tools into educational frameworks, thereby enhancing student engagement and learning experiences. This research lays a foundation for future exploration of the integration of GenAI technologies in education and provides a framework for considering their potential impacts on learning outcomes, student well-being, and the broader educational landscape.

### 5.2 Limitations of the study

This study acknowledges several limitations. First, the research sample is limited to university students from Southwest China, which restricts the generalizability of the findings to broader populations. Future research should include a more diverse sample to increase the applicability of the results. Second, the study relies on self-reported data, which may be subject to response bias and might not fully reflect users’ actual behaviors and experiences. Future studies could incorporate objective metrics and observational data to gain a more comprehensive understanding of user acceptance and usage patterns. Third, the cross-sectional design limits causal inferences, as both behavioral intention and actual behavior were measured concurrently. Future research could address this limitation by conducting longitudinal studies or follow-up surveys to track changes in behavior and intentions over time. Additionally, due to space constraints, this study focused primarily on the analysis and application of the 2SSX framework, limiting the comprehensiveness of the analysis. Further exploration, such as subgroup or moderation analysis, could provide a more detailed understanding of the factors influencing GenAI adoption.

### 5.3 Future research directions

Drawing from the conclusions of this study, future research could explore several important avenues: First, it is crucial to examine the long-term effects of GenAI tool usage on users’ learning outcomes and research performance. Investigating how GenAI tools integrate into the educational process and their impact on students’ academic achievements could provide significant insights into their sustained effectiveness. Second, exploring the role of user training and support systems in enhancing acceptance and addressing usage concerns is vital. Developing effective training programs and support structures will be essential for ensuring the successful integration of GenAI tools within educational environments. Third, examining the influence of personalized feedback and adaptive features in GenAI tools could improve their efficiency as supplementary learning aids. This line of research could focus on how tailored content and real-time adjustments might enhance student engagement and learning outcomes. Finally, it is important to investigate the ethical and societal implications of GenAI usage, including concerns related to bias, privacy, and algorithmic accountability. Addressing these issues is key to ensuring the responsible and equitable application of GenAI technologies in academic settings.

## Data Availability

The original contributions presented in the study are included in the article/supplementary material, further inquiries can be directed to the corresponding author.
